# H_3_PO_4_/KOH Activation Agent for High Performance Rice Husk Activated Carbon Electrode in Acidic Media Supercapacitors

**DOI:** 10.3390/molecules28010296

**Published:** 2022-12-30

**Authors:** Nasser A. M. Barakat, Osama M. Irfan, Hager M. Moustafa

**Affiliations:** 1 Chemical Engineering Department, Faculty of Engineering, Minia University, Minya 61519, Egypt; 2 Department of Mechanical Engineering, College of Engineering, Qassim University, Buraydah 51452, Saudi Arabia

**Keywords:** activated carbon, supercapacitors, rice husk, chemical activation

## Abstract

H_3_PO_4_/KOH combined solution is proposed as a new effective activation agent for activated carbon production from rice husk. Several activated carbon samples were produced by using different volumes of the utilized acid and alkali individually, in addition to the combined solution. FTIR results indicated that the mixed agent partially decomposed the chemical compounds on the rice husk char surface, resulting in an increase in the surface area. Moreover, XRD and EDS analyses showed the presence of a considerable amount of amorphous silica. Electrochemical measurements concluded that the volume of the activation agent solution should be optimized for both single and mixed activation agents. Numerically, for 0.3 g treated rice husk char, the maximum specific capacitance was observed at 7, 10 and 14 mL of H_3_PO_4_, KOH (3 M) and mixed (1:1 by volume) activation agents, respectively; the determined specific capacitance values were 73.5, 124.2 and 241.3 F/g, respectively. A galvanostatic charging/discharging analysis showed an approximate symmetrical triangular shape with linear voltage versus time profile which indicates very good electrochemical performance as an electrode in the supercapacitors application. The stability of the proposed activated carbon was checked by performing a cyclic voltammetry measurement for 1000 cycles at 2 mV/s and for 30,000 cycles at 10 mV/s. The results indicate an excellent specific capacitance retention, as no losses were observed.

## 1. Introduction

Because of its high power density, fast charge/discharge rates, large storage capacity, dependability, and long lifetime, supercapacitors have led the way in energy storage technology in recent decades [1]. In the last decade, supercapacitors have been at the heart of energy storage technology [2]. However, achieving the higher needs of the future systems such as portable electronics, hybrid vehicles, and large-scale industrial equipment requires the development of novel materials and a greater knowledge of electrochemical interfaces at the nanoscale [3,4].

One of the core elements that determine supercapacitors’ performance is the electrode material, which has sparked a lot of research interest. Transition metals (such as NiO, RuO_2_, MnO_2_, and IrO_2_), carbon-based materials (such as carbon black, carbon nanotubes, glassy carbon, and activated carbon), and conducting polymers are some of the most often used electrode materials [5,6,7,8]. In the last 10 years, research has emphasized the discovery and development of green carbon materials, with a particular focus on those having the ability to minimize agro-industrial waste; such as activated carbon (AC).

The conversion of renewable biomass to ACs is considered as more worthwhile when considering production costs and energy/environmental effects [9]. Until now, ACs have been synthesized from various renewable biomasses, such as rice husk [10,11], cellulose [12,13,14], lignin [15,16] and others. Compared to other agricultural wastes, rice husk is produced in high amount and should be disposed of in an environmentally safe manner. Rice husks, often known as “rice hulls”, are the outer shells that surround the rice kernels. These components are one of the rice processing’s agricultural wastes. Around 571 million tons of rice are produced annually in the world, yielding approximately 140 million tons of useless rice husk. As a result, utilizing this renewable resource in a valuable product through a cost-effective activation processes is highly recommended from both an industrial and environmental point of view [17]. Unfortunately, in many places, this agricultural waste is burned or discarded as trash or as fertilizer. Consequently, in several countries, rice husks become a source of pollution due to the uncontrolled burning operations [18,19,20]. Paradoxically, rice husks could be one of the most readily available agricultural resources. Recently, rice husks have been investigated to produce effective electrodes, through graphitization and specific activation processes for supercapacitor applications [21,22].

Carbonization and activation, which can be done separately in a two-stage process or combined in a single stage one, are typically used in the preparation of activated carbon. In graphitization, the precursor is carbonized at a low temperature, usually between 400 °C and 850 °C, in the absence of oxygen. Activation, the method of changing carbonaceous materials into activated carbon, includes physical activation, chemical activation, combined chemical and physical (physiochemical) activation, and microwave-induced activation [23].

Among the aforementioned activation techniques, chemical activation drew the most attention in both research and industrial scales due to its lower activation temperature and higher yield. In this regard, several chemicals were investigated as activation agents such as KOH, ZnCl_2_, H_3_PO_4_, K_2_CO_3_, NaOH, HCl, etc. However, potassium hydroxide (KOH) and ortho phosphoric (H_3_PO_4_) are the most commonly chosen agents because of their efficient performance, in addition to the fact that they can be easily removed after activation of the carbon by washing by hot and cold water [20,24,25,26,27,28].

KOH is widely used due to the distinguished improvement in the surface area electrochemical performance produced ACs. The surface area increases distinctly upon utilizing of KOH due to the formation of some gases during the heating of KOH with carbon resulting in the formation of numerous amounts of micro and macro pores [29,30]. In addition, the interaction of metallic potassium in the carbon matrix extends the space between carbon atomic layers, resulting in an increase in total pore volume [31,32].

Likewise, phosphoric reveals an interesting performance as an activating agent due to its high tendency to attach with the rice husk and its subsequent decomposition [33]. As a result, phosphoric acid works as a catalyst for the depolymerization of the lignocellulosic precursor’s constituents into smaller units. Phosphoric acid generates phosphate and polyphosphate bridges that bind biopolymer fragments after chemical activation of lignocellulosic precursors, preventing material contraction due to temperature effects. As a result, the material’s mechanical characteristics are improved [27,34]

However, to the best of our knowledge, these two effective activation agents were not investigated together. The objective of this study is to investigate the exploiting of a combined mixture from KOH and H_3_PO_4_ in preparation of activated carbon from rice husk and investigate the electrochemical characteristics of the produced AC and its adequacy as an electrode in supercapacitors. Interestingly, it was found that the combination between these two activation agents led to produce an efficient AC for supercapacitors applications compared to utilizing an individual agent; however, the mixing ratio has to be optimized to get the best performance.

## 2. Results and Discussion

### 2.1. Activated Carbon Characterization

**Figure 1:**
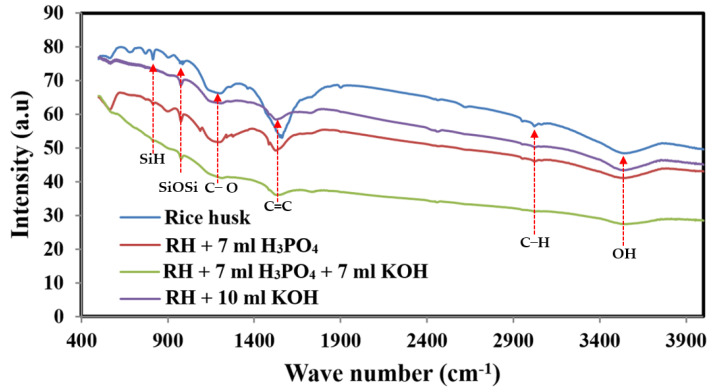
FTIR spectra of prepared sample RH, RH + 7 mL H_3_PO_4_, RH + 10 mL KOH and RH + 7 mL H_3_PO_4_: 7 mL KOH.

Figure 1 shows FTIR spectra of the pristine and activated carbon obtained from rice husk carbonization. At 3453 cm^−1^, -OH stretching vibration of the –SiOH group is observed. Within a wavelength region of 2960–2873 cm^−1^, the aliphatic C–H groups stretch asymmetrically and symmetrically [35]. Although in the untreated carbonized rice husk, the representative peak of the C-H stretching appears very small, this peak diminishes in the KOH and H_3_PO_4_–activated carbon and completely disappeared in the combined agent-activated carbon. Saturated carbon-hydrogen bonds have low adsorption affinity, so it is expected that the chemically treated carbonized carbon will have high ion electrosorption. Si–O–Si stretching occurs at 1108 cm^−1^ and overlaps with phosphates formed by phosphoric acid activation, whereas Si–H stretches are observed at 795 cm^−1^. As a result, the presence of these bands is restricted to phosphorous and phospho-carbonaceous chemicals [27,36]. Peaks appearing in the 1400–1600 cm^−1^ range suggest the presence of C=C bonds in aromatic compounds [37,38]. It is clear that all the previously assigned peaks are obtained with all formulations with different intensities. For instance, the peak representing C=C bonds (in the range of 1400–1600 cm^−1^) possesses the maximum intensity in the spectrum of the untreated rice husk char. However, this peak displays lower intensities with the treated samples. Comparatively, the treated sample by the phosphoric acid agent shows the highest intensity, which indicates the protecting of more C=C containing compounds from cracking during the activation process. A similar conclusion can be drawn for the Si-O-Si peak (1108 cm^−1^). Typically, the highest intensities are accomplished with the untreated and phosphoric agent samples. In general, for the treated samples, the lowest peak intensities are corresponding to the combined-agent treated sample and the highest ones are related to the phosphoric acid-treated activated carbon. This finding suggests extensive dissolution of the chemical compounds in case of utilizing the combined agent in the activation process, which might be assigned as a reason behind the observed good electrochemical characteristics of this sample. In other words, as the capacitance characteristic is based on the formation of electric double layer, the function groups’ nature and intensity strongly affect the capacitance performance. Consequently, the non-activated sample shows the lowest specific capacitance activity. However, the best and worst performances were related to the samples activated by the combined and acid agents, as will be discussed later.

**Figure 2:**
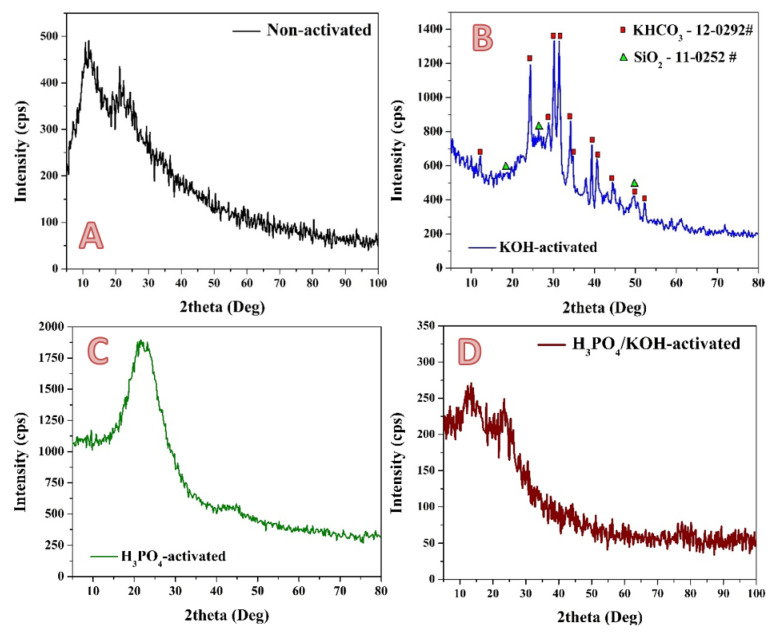
XRD patterns of non-activated; (**A**), and activated by KOH only; (**B**), H_3_PO_4_ only; (**C**) and combined H_3_PO_4_–KOH solution; (**D**) rice husk carbonized powder. The used solution was 14 mL.

XRD analysis (Figure 2) was carried out to check the chemical composition of the prepared pristine (non-activated) and activated samples using KOH, H_3_PO_4_ and the proposed combined system solutions. It is known that the rice contains a considerable amount of silicon compounds, which is also supported by FT IR results (Figure 1). Accordingly, there are some reports that were introduced to extract silica from this agricultural waste [39,40,41]. In the prepared graphite, the presence of amorphous silica could be indicated due to appearance a wide peak at 2*θ* value of 21.9° corresponding to (101) crystal plane of *cristobalite* phase (# 39-1425). On the other hand, the broad beak at 2*θ* value ~12° corresponding to d_001_ plane confirmed the oxidation of the graphitic structure and the formation of oxygenated functional groups such as carbonyl, carboxyl, epoxide and hydroxyl groups in the prepared activated carbon [42]. Therefore, from FTIR and XRD results, it can be concluded that the surface of the non-activated sample contains high amounts from the oxygenated groups (COOH, OH, C–O and C–O–C) because of the using of a relatively low calcination temperature of 600 °C. This finding is consistent with other reports [43].

Alkaline activation is widely used and shows good performance. The previous reports stated that KOH can react with carbon and form new chemical compounds [29,30,44]. This hypothesis was confirmed in the present study, as shown in Figure 2B. The XRD pattern of the KOH-treated sample indicates that the produced activated carbon contains KHCO_3_ compound. The appearance of strong diffraction peaks at two theta values of 12.0°, 24.2°, 28.8°, 30.0°, 31.2° and 34.1° corresponding to (200), (400), (201), (−311) and (211) crystal plans indicates the formation of Kalicinite (#12-0292). The diffusion and intercalation of KOH into the carbon lamellae is a crucial factor to take into account when analyzing the effects of potassium in the activation process [45]. However, in contrast to several previous reports which utilized KOH as an activation agent at high treatment temperatures [46], the activation process in this study has been performed using a reflux system. Therefore, the formation of KHCO_3_ is reasonable. In detail, when the KOH is used, it undergoes dehydration (at 400 °C) during the conventional thermal activation. Removal of water leads to separation of the carbon lamellae. Accordingly, a part of the potassium forms alkalides such as –OK groups and K_2_CO_3_ [47]. Silica was also detected in the sample, as it is indexed in the figure. Potassium hydroxide reacts with silicon dioxide to produce potassium metasilicate, potassium metatetrasilicate, and water. This reaction takes place at a temperature of 900–1000 °C. However, alkali reflux treatment showed strong power in removing the silica, as can be concluded from the small detected amount of SiO_2_ in the produced activated car from the XRD analysis. It is known that the acidic groups in the prepared activated carbon can be neutralized by various bases [48]. Therefore, the appearance of the representative intensity peak of the oxygenated activated carbon after the activation process using KOH solution could be attributed to the neutralization of many oxygenated groups during the activation step. This hypothesis is supported by Wenwen Zhao et al, who studied the neutralization of graphene oxide using various chemicals [49]. In that work, it was concluded that neutralization of the oxygenated groups results in decreasing the intensity of the graphene oxide peak in the XRD pattern.

**Figure 3:**
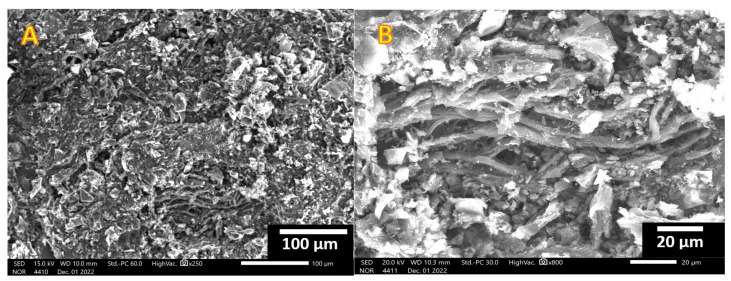
Low; (**A**) and high; (**B**) SEM magnifications for the obtained rice husk activated carbon using KOH/H_3_PO_4_ combined activation agent.

Figure 3B displays the XRD pattern of the phosphoric acid-treated sample. The broad fuzzy peaks C(002) at 2*θ* = 15°–30° indicate that the carbon has mainly an amorphous structure [50]. On the other hand, the low and wide C(100) diffraction peaks (2*θ* = 40°–50°) are related to the *α* axis of the graphite structure, which may indicate that the tested materials contain a number of graphite planes [51,52].

**Figure 4:**
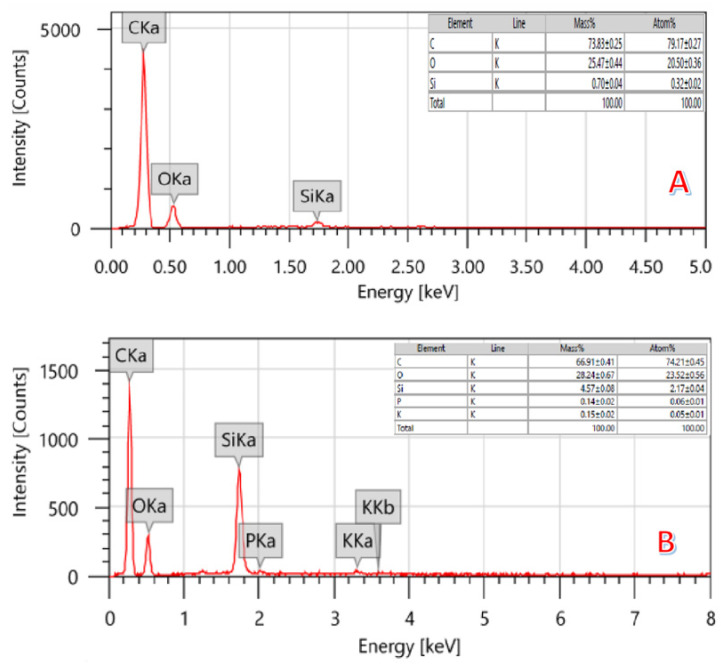
EDS of activated (**A**) (by 14 mL H_3_PO_4_-KOH solution) and pristine (**B**) carbon obtained from graphitization of the rice husk.

Using the combined agent in the activation process resulted in producing an activated carbon with different structures compared to both of pristine and individual medium activation agent (i.e., acid and base agents), as shown in Figure 4D. As shown in the figure, although KOH is included in the activation process, no inorganic compounds were detected in the produced carbon. Moreover, the high intensity peak appeared in the acid-treated sample was detected with comparatively small intensity upon utilizing the combined agent. Furthermore, the combined agent could partially neutralize some functionalized activated carbon groups, which was reflected in a decrease in the representative peak intensity. Accordingly, it is clear that the surface structure of the produced activated carbon after exploiting the KOH/H_3_PO_4_ combined agent is considerably different than the KOH and H_3_PO_4_-treated carbons. Consequently, a different electrochemical performance was observed.

Two magnification SEM images for the prepared activated carbon using the combined agent are displayed in Figure 3. As is shown, the obtained powder consists of thin flakes of activated carbon, which reflects the high surface area.

Figure 4 depicts the regular EDS of the treated (by the combined agent; Figure 3A) and pristine (Figure 3B) graphite obtained from the rice husk. The inset table in each panel shows the results of the elemental analysis performed on AC samples. As is shown, the carbon content in the activated sample (79.17 ± 0.27) is higher than the non-treated one (74.21 ± 0.45), which can be attributed to the removal of some pollutants during the activation process. This hypothesis can be supported by decreasing the silicon content in the treated sample. Moreover, as was concluded from XRD results, some functional groups disappeared during the activation process. Therefore, it is expected that the removed pollutants left nanopores behind, which enlarges the total surface area. The thermal combustion of rice husk produces a porous nanomatrix.

**Figure 5:**
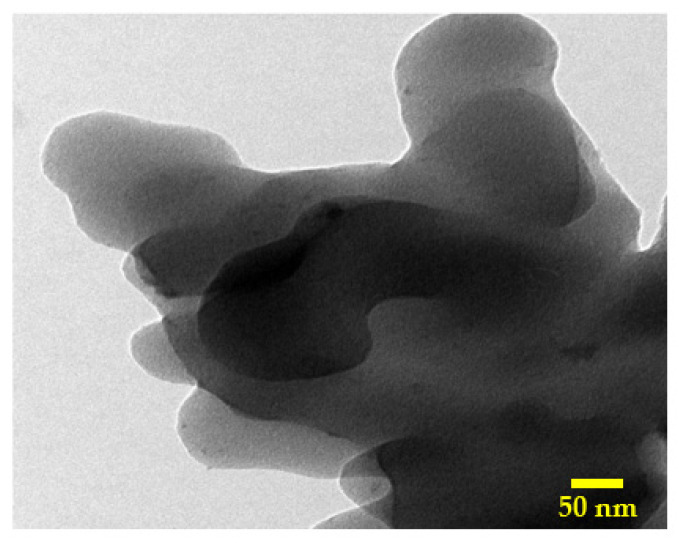
TEM image for the obtained activated carbon using H_3_PO_4_/KOH mixture (14 mL sample).

Figure 5 depicts the TEM image for the activated carbon using the combined activation agent (14 mL sample). As shown in the figure, the obtained activated carbon is close to multilayer graphene oxide, as the thickness is not high. Moreover, some small black dots can be observed, which can be attributed to the silica nanoparticles.

**Figure 6:**
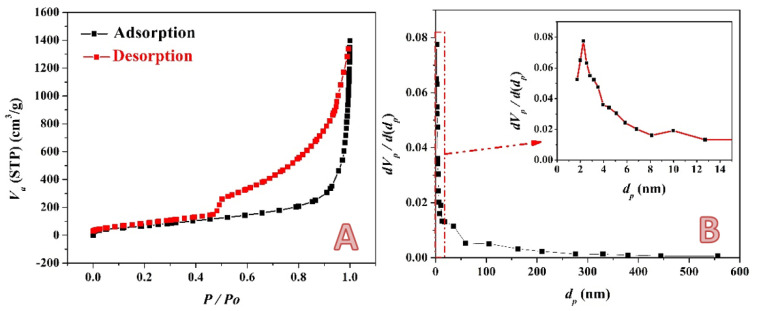
Nitrogen adsorption/desorption isotherm; (**A**) and pore size distribution curve; (**B**) for the prepared activated carbon using the combined activation agents (14 mL sample).

The nitrogen adsorption-desorption isotherm of the prepared activated carbon using the combined activation agents (14 mL sample) is represented in Figure 6A. As shown, the figure displays a typical IV type with a hysteresis loop at a relative pressure ranging from 0.48 to 0.98. The hysteresis loop indicates the existence of mesopores. Moreover, the hysteresis loop concludes the presence of capillaries with necked openings [53]. Additionally, a small increase in nitrogen absorption is seen at low relative pressures, suggesting the development of some micropores. The Nonlocal Density Functional Theory (NLDFT) approach is used to derive the pore size distribution (Figure 6B) from the isotherm’s adsorption branch. The outcome shows that the activated carbon that was created utilizing all of the activation chemicals had large pores, measuring between 2 and 3 nm. Because of the activation of the KOH/H_3_PO_4_ system, activated carbon has well-developed mesopores that are bigger than 3 nm as well. Moreover, the BET surface area and total pore volume of the produced activated carbon are 325.67 m^2^/g and 1.8145 cm^3^/g, respectively. Moreover, the surface area of the acid-activated carbon was investigated; the results indicated that the BET-specific surface of this sample is 181.1 m^2^/g.

**Figure 7:**
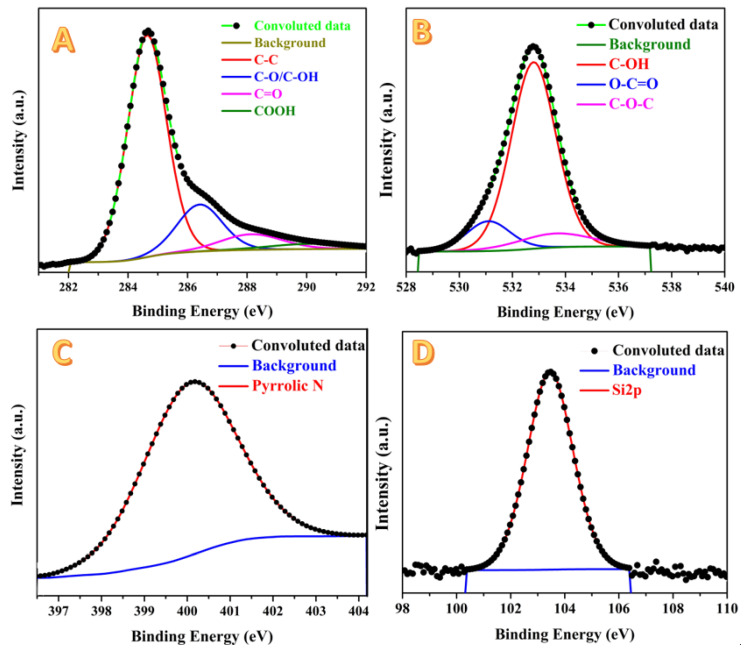
XPS analysis results for H_3_PO_4_/KOH combined agent-activated carbon: C1s; (**A**), O1s; (**B**), N1s; (**C**) and Si2p; (**D**).

Figure 7 displays XPS analyses for the prepared activated carbon using the combined agent in the chemical activation process. As shown in Figure 7A, in the spectra of the C 1s region, the peaks were de-convoluted into four chemically shifted segments [54,55]. The first segment at 284.7 eV could be assigned to C–C/C–H non-oxygenated [55,56]. The interaction of carbon atoms with oxygen in either hydroxyl (C–OH) or epoxide (C–O) functional groups is detected at 286.5 eV in the second segment. The carbonyl (C=O) can be identified in the third segment, at 288.3 eV. Finally, the presence of the carboxyl group (COOH) can be claimed from the fourth segment at 290.2 eV [57]. As shown in Figure 7B, three segments are observed in the de-convolution of the peak in the O 1s region, namely: C=O (oxygen double bond to aromatic carbon) at 531.1 eV, C–O (oxygen single bond to carbon) at 533.8 eV, and C-OH (carbon single bond to the hydroxyl group) at 532.5 eV [58]. The nitrogen element is a primary constituent in several kinds of biomasses including rice husk. This hypothesis was confirmed by the XPS analysis as shown in Figure 7B. Typically, the surface of the investigated sample contains pyrrolic nitrogen due to the appearance of a single peak at 400.2 eV that perfectly matches the literature at 532.5 eV [58]. Silicon is a characteristic element for the rice husk, so silica was extracted from this biomass [59]. Accordingly, an XPS analysis revealed the existence of an Si element in the investigated activated carbon, as shown in Figure 7D. Typically, at the Si 2p region, a predominant peak at 103.5 eV is observed, representing the Si 2p orbital.

### 2.2. Electrochemical Measurements

The type, content, and concentration of the electrolyte is crucial for supercapacitor performance, much as the electrode materials. A wide voltage window, excellent electrochemical stability, high ionic concentration and conductivity, low viscosity, and low toxicity are the required characteristics of a good electrolyte. There are three categories of common electrolytes: aqueous, organic liquid, and ionic liquid. Aqueous electrolytes have a low internal resistance, a high ionic conductivity, and low cost compared to the other types. The currently employed aqueous electrolytes can be classified into three types: acidic solution (such as H_2_SO_4_ solution), alkaline solution (such as KOH solution), and neutral solution (such as Li_2_SO_4_, Na_2_SO_4_, or KCl solution). Sulfuric acid is the most frequently used aqueous acid electrolyte in supercapacitors simply because of its extremely high conductivity (~0.8 cm^−1^ at 25 °C for 1 M H_2_SO_4_) [60,61]. The ideal molar concentrations to achieve the best attainable ionic conductivities of a given electrolyte at a given temperature have already been explored, since the ionic conductivity of an electrolyte depends on concentrations. So far, electrolyte ionic conductivity can be quickly dropped when the concentration becomes extremely low or excessively high. Researchers have found that the specific capacitance attained in the H_2_SO_4_ electrolyte is significantly greater than the specific capacitance in a neutral electrolyte for carbon-based capacitors [60]. Additionally, compared to the neutral electrolyte, the ESR value of supercapacitors is substantially lower because of the strong ionic conductivity of H_2_SO_4_ [62]. Accordingly, in this study, sulfuric acid (0.1 M) has been selected to be used as an electrolyte.

To properly investigate the influence of the activation agent medium, the graphitized rice husk has been treated by three media; phosphoric acid, potassium hydroxide (3 M) and equi-volume H_3_PO_4_/KOH (3 M) mixed agents solutions. Moreover, every solution has been used in different volumes added to a fixed amount of rice husk activated carbon of 0.3 g.

**Figure 8:**
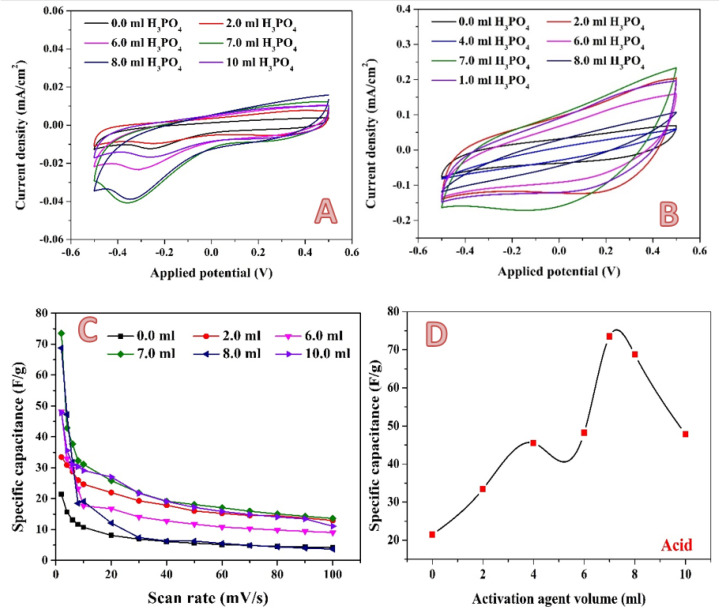
Cyclic voltammograms in the presence of 0.1 M H_2_SO_4_ electrolyte at a scan rate of 2 mV; (**A**) and 100 mV/s; (**B**) for the 0.3 g activated carbon obtained from rice husk using different volumes of phosphoric acid. Influence of the scan rate on the specific capacitance; (**C**) and effect of the phosphoric acid volume on the specific capacitance (at 2 mV/s); (**D**).

For the acidic media, the CV curves of the pristine and activated samples at 2 and 100 mV/s are shown in Figure 8A,B, respectively. As shown, the treated AC was shown to have a quasi-rectangular form for CV curves, which is typical of electrical double layer capacitance. Furthermore, no significant redox signal was seen for all CV curves, indicating the absence of pseudo-capacitance in the treated AC samples, and indicating that electrochemical double layer capacitance is the major charge storage mechanism in the prepared AC samples. The absence of pseudo-capacitance in AC could be due to the structure’s insignificant oxygen functional groups, which could lead to the formation of the faradaic reactions [4,63]. By integrating the area under the curve for the average value, the specific capacitance values of samples were derived from CV curves [64,65]; Figure 8C. As can be seen from the figure, for all samples, the specific capacitance decreases with the increasing the scan rate. The current generally rises with an increase in the scan rate, while specific capacitance decreases. It is thought that slowing the scan rate can enable the electrolyte to enter pores more thoroughly and to make greater contact with the internal surface of the electrode material. As a result, more charge is believed to be stored on the surface of the electrode, increasing the measured capacitance and bringing it closer to the intrinsic capacitance. In contrast, low capacitance is detected at high scan rates because the electrolyte and electrode are in contact for a very short time as a result of which less charge is stored on the electrode surface, and therefore a low capacitance is observed [66,67]. Figure 8D represents the influence of phosphoric acid volume on the obtained specific capacitance at a 2 mV/s scan rate. As is shown, the relationship is not linear. Instead, the highest specific capacitance is observed at approximately 7 mL acid. In activation by phosphoric acid, first the acid is adsorbed onto the surface, and then the decomposition of the acid results in the formation of micro and macro pores. Li et al. stated that phosphoric acid acts as an acid catalyst bond cleavage reaction and as a reactant in the formation of crosslinks via the crystallization and condensation process [68]. Moreover, the acid can combine with the organic functions to form phosphate and polyphosphate bridges that connect and crosslink material fragments [50]. As an analogy to the general features of the chemical reactions, the reactant(s) concentration is a highly effective parameter in the reaction yield. Accordingly, it can be claimed that increasing the acid volume more than the optimum value (7 mL) may lead to the blocking some pores, which negatively affects the electrochemical characteristics.

**Figure 9:**
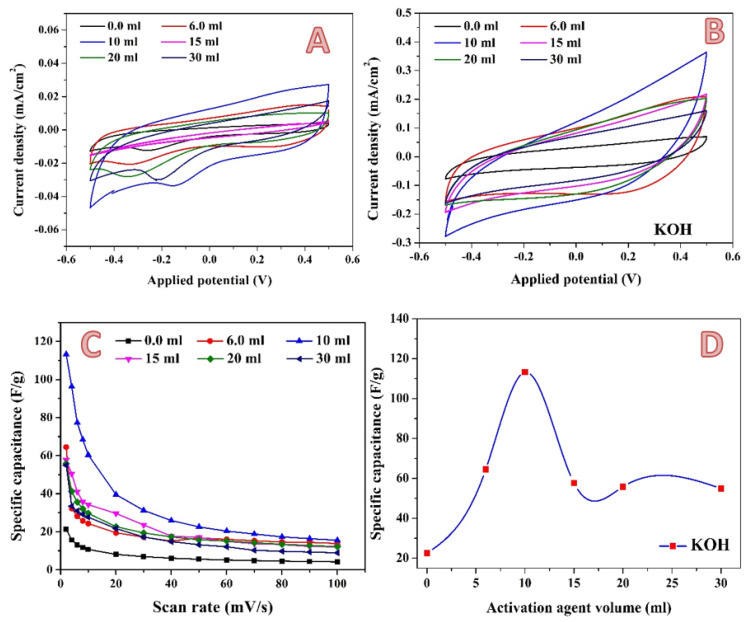
Cyclic voltammograms in the presence of 0.1 M H_2_SO_4_ electrolyte at a scan rate of 2 mV; (**A**) and 100 mV/s; (**B**) for the 0.3 g activated carbon obtained from rice husk using different volumes of 3.0 M KOH solution. Influence of the scan rate on the specific capacitance; (**C**) and the effect of the alkaline solution volume on the specific capacitance (at 2 mV/s); (**D**).

In addition to phosphoric acid, potassium hydroxide has also been widely utilized as an activation agent. Moreover, it was utilized at a commercial level. For instance, the Amoco Corporation has utilized KOH for converting aromatic precursors such as coal and petroleum coke into high surface area carbons (around 3000 m^2^/g). The Kansai Coke and Chemicals Co. obtained a license to use the method on a pilot plant after it was commercialized in the 1980s. The large surface area of the activated carbon is caused by its mostly microporous structure, and its total pore volume is very high: 2.0–2.6 mL/g [69,70]. Figure 9A,B show the cyclic voltammograms at 2 mV/s and 100 mV/s for carbon activated by different amounts of 3 M KOH solution. Resembling the phosphoric acid activation, as in the case of KOH, no redox peaks could be seen in the CV results, which indicates absence of pseudo-capacitance in the investigated samples. As previously discussed, the specific capacitance is inversely proportional with the scan rate, as shown in Figure 9C. The increase in scan rate limited the ions’ accessibility to the electrode surface. This phenomenon could be related to the ion sieving effect, where the ultrafine pores in treated AC could only be accessed at a slow diffusion rate [71,72]. Compared to H_3_PO_4_-activated carbon, which reveals a highest specific capacitance value of 73.5 F/g at 7 mL utilized solution, the maximum specific capacitance in the case of utilizing KOH as an activation agent is higher. As shown in Figure 9D, a highest specific capacitance value of 113.24 F/g is obtained when the volume of the used alkali solution was 10 mL. However, a greater increase in the activation agent solution volume does have a negative impact on the specific capacitance.

**Figure 10:**
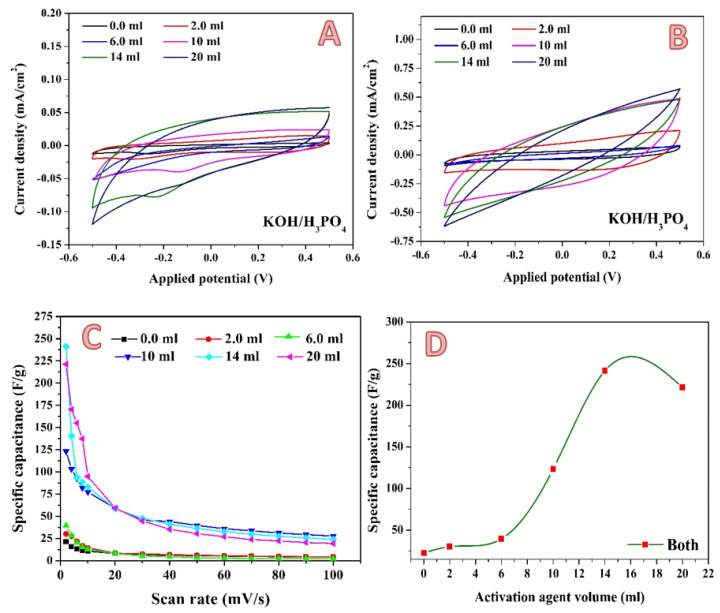
Cyclic voltammograms in the presence of 0.1 M H_2_SO_4_ electrolyte at a scan rate of 2 mV; (**A**) and 100 mV/s; (**B**) for the 0.3 g activated carbon obtained from rice husk using different volumes of KOH/H_3_PO_4_ mixed activation agent. Influence of the scan rate on the specific capacitance; (**C**) and effect of the alkaline solution volume on the specific capacitance (at 2 mV/s); (**D**).

A newly proposed combined activation agent was introduced. As could be concluded from exploiting the phosphoric acid and KOH solution individually, the optimum volume is close, at 7 and 10 mL for the acid and base, respectively. Therefore, the two activation agents have been mixed in a volume ratio of 1:1. Figure 10 shows the electrochemical characteristics of the produced activated carbon. Cyclic voltammetry measurements have been performed at different scan rates in the presence of 0.1 M H_2_SO_4_. Figure 10A,B display the results obtained at 2 and 100 mV, respectively, as examples. In contrast to the previous results, in the case of the combined activation agent, an observable jump in the specific capacitance is seen when the activation agent volume increased from 6 to 10 mL, as shown in Figure 10C. Moreover, using the combined activation agent results in a strong increase in the maximum specific capacitance. As seen in Figure 10D, a specific capacitance of 241.3135 F/g was obtained when 14 mL of combined activation agent was utilized. This value is more than two- and three-fold greater when compared to KOH (113.5 F/g) and H_3_PO_4_ (73.5 F/g), respectively, which is considered as significant progress. This finding can be ascribed to the unique micro/mesoporous structure of the sample, the abundant surface active sites for adsorption–desorption within micropores, fast ionic transportation channels within the mesopores, and the short diffusion distance of electrolyte ions from mesopores to micropores [73].

In the combined activation agent, the reaction between the acid and base is not simple due to the possibility of formation of different products; KH_2_PO_4_, K_2_HPO_4_ and K_3_PO_4_. Moreover, some compounds are not stable during the thermal treatment process. For example, the potassium orthophosphate can decompose to potassium pyrophosphate by losing half a mole of water according to the following reaction [74]:

2KH_2_PO_4_ → K_2_H_2_P_2_O_4_ + H_2_O (1)

Moreover, this newly formed potassium salt can show a further phase change to form potassium metaphosphate by losing another half mole of water, as follows [74]:

K_2_H_2_P_2_O_4_ → 2KPO_3_ + H_2_O (2)

It is worthy of mention that 1.9 mL of the used KOH solution was required to neutralize the used 7 mL phosphoric acid when a titration analysis was performed using methyl orange as indicator. Therefore, it can be claimed that there is excess KOH solution in addition to the formed potassium phosphate salts in the used mixed agent. The sophisticated reaction between H_3_PO_4_ and KOH led the authors to investigate this combined agent which has several compounds in a single solution in the activation of the rice husk carbonized product. In other words, it can be claimed that the observed distinct performance of the combined activation agent may be attributed to the various produced salts formed in the solution, which can result in different reaction affinities with the chemical compounds existing on the graphite surface.

Table 1 shows a comparison between activated carbons prepared from RH using different activation strategies that have been investigated at different electrolytes. As shown in the table, although the used electrolyte concentration is low (0.1 M H_2_SO_4_) and the used activation strategy is simple, the obtained specific capacitance is high compared to many reported activated carbon obtained from the rice husk. It is worth mentioning that there is a peak that appears in the reduction direction only at the low scan rate with all used activation agents. This peak does not indicate a redox capacitance because this peak first appears in a half cycle only, so it cannot be considered as a complement of a redox pair. Moreover, it appears at the low scan rate only. This peak might represent the reduction of an impurity on the electrode surface.

**Figure 11:**
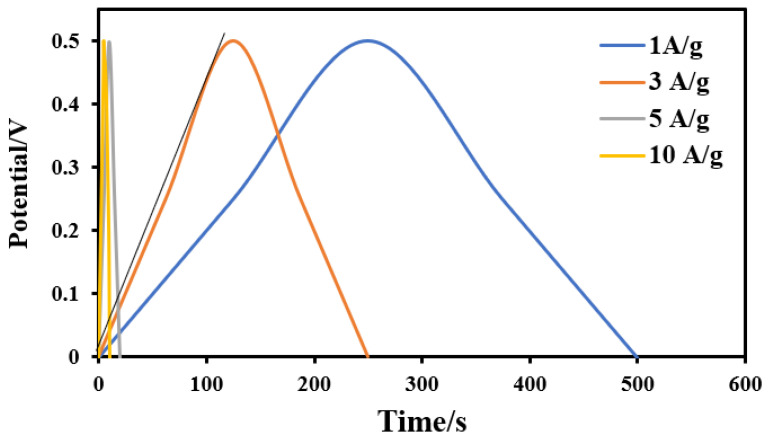
Galvanostatic charge/discharge curves for the activated carbon electrode obtained from activation by a combined H_3_PO_4_/KOH activation agent (14 mL) at current densities of 1, 3, 5 and 10 A/g.

The electric double layer capacitance (EDLC) was tested at constant current charge/discharge regimes (between 1 and 10 A/g) within the voltage range from 0 to 0.5 V in 0.1 M H_2_SO_4_. The galvanostatic charging/discharging analysis (GCD) is an important electrochemical for the supercapacitors electrodes. Figure 11 displays the GCD curves of the sample showing the maximum specific capacitance (activated by 14 mL combined activation agents) at different current densities; 1, 2, 5 and 10 A/g. It can be observed that the charge–discharge curves of the prepared activated carbon electrode are of an approximately symmetrical triangular shape with a linear voltage versus time profile. The introduced activated carbon from rice husk shows maximum discharge times, either at high or low current densities, indicating the best electrochemical performance [87]. Moreover, the nearly symmetric triangle shape of the obtained charge and discharge curves indicates that the proposed electrode has high reversibility at high current loadings [88]. In addition, only a slight *IR* drop is observed at high current loading, which reveals the high rate capability and small internal resistance [89]. For the other samples, the measurements have been conducted and similar behaviors were obtained for both acid and alkali–activated samples (data are not shown). As mentioned in the CV discussion, the capacitance of the introduced activated carbon cannot be considered as redox capacitance. Therefore, based on CV and GCD results, it can be claimed that the capacitance is related to electric double layer capacitance.

The galvanostatic specific capacitance can be estimated from the following equation:

(3) $$C=\frac{I}{m}\times \frac{\Delta t}{\Delta V}$$

where *I/m* is the current density per unit mass of the used materials (F/g), Δ*V* is the potential drop during the discharge step (V), and Δ*t* is the required time to complete full discharge (s). Accordingly, for the combined agent-activated sample, the specific capacitance at lowest current density (1 A/g) was determined to be ~500 F/g, where at the highest values (5 and 10 A/g) it decreases and becomes independent on the assigned current density, as it was 99.5 F/g. It is worth mentioning that the gravimetric capacitance values are calculated from CV and GCD bases and that usually there are differences between the obtained values [90].

**Figure 12:**
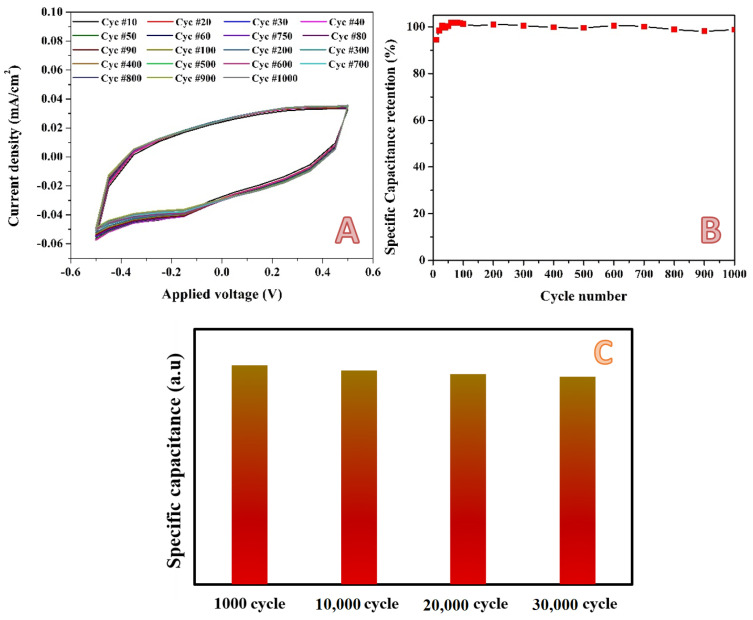
Cyclic voltammetry (selected cycles from 1000 cycles) for the electrode obtained from activation using H_3_PO_4_/KOH combined solution (14 mL) in 0.1 M H_2_SO_4_ at scan rate 2 mV/s and 25 °C; (**A**), and specific capacitance retention; (**B**). Panel (**C**) displays the specific capacitance retention after 30,000 cycles within a potential window of −0.2 to 0.2 V (vs. Ag/AgCl) at a scan rate of 10 mV/s using 0.1 M H_2_SO_4_ electrolyte.

Working in low concentration acidic media is highly susceptible because of the high corrosion power of these media, especially for the metal–containing electrodes. Consequently, the stability is a key factor of the proposed electrodes. To properly investigate the stability of the introduced activated carbon, a cyclic voltametry measurement was carried out for 1000 successive cycles at a scan rate of 2 mV/s for the best electrode in the presence of 0.1 M H_2_SO_4_ electrolyte within a potential window of −0.5~0.5 V (vs. Ag/AgCl electrode). As shown in Figure 12A, almost no change in the formed cycles can be observed, which reveals excellent stability for the proposed electrode. Consequently, very distinguished specific capacitance retention could be obtained, as shown in Figure 12B. There was almost no loss in the specific capacitance after 1000 cycles. This indicates that the charge/discharge process of the investigated electrode is highly reversible, which benefits from the good conductivity of the electrode and the stable carbon skeleton [91,92]. Moreover, the excellent cycling stability is inseparable from the stable skeleton structure of the cellulose component and the high conductivity of the cellulose derived porous carbon. Numerically, the observed specific capacitance retention was more than 95% after 1000 cycles at scan rate of 2 mV/s. The stability has been examined by applying the cyclic voltammetry process for 30,000 cycles within a potential window of −0.2~0.2 V (vs. Ag/AgCl) at a scan rate of 10 mV/s using the same electrode and electrolyte. As shown in Figure 12C, there is also a very good stability specific capacitance retention, even for 30,000 successive cycles.

## 3. Materials and Methods

### 3.1. Materials

Ortho phosphoric acid and potassium hydroxide (Sigma Aldrich, Saint Louis, MO, USA) were of analytical grade and were used without further purification. Rice husk samples were collected from local farms in EL-Minya province, Egypt.

### 3.2. Preparation of Activated Carbon from Rice Husk

The husk was washed and dried at 110 °C for 24 h. The samples with moisture content lower than 5% were selected for further experiments. The selected rice husk material was ground and sieved to collect mesh particles.

#### 3.2.1. Carbonization Step

The cleaned and dry rice husk material was carbonized at 600 °C in a furnace for 2 h. After carbonization, the obtained rice husk char (RH) was cooled down to room temperature in a desiccator.

#### 3.2.2. Activation Step

The activation step was performed by the reflux mode activation process using KOH and H_3_PO_4_ solutions individually and together in different amounts. The treated amount of the carbonized rice husk was fixed at 0.3 g for all experiments. To ensure a proper reaction between the activation agent and the rice husk, the rice husk char/activation agent suspension was refluxed for 3 h. Different volumes of H_3_PO_4_ (stock; 2, 4, 6, 8 and 10 mL) and KOH (3 M concentration; 4, 10, 20 and 30 mL) were used in the individual investigation. For the combined activation agents, an equal volume form H_3_PO_4_ stock and KOH aqueous solution (3 M) were mixed and used as an activation solution. Different volumes from the mixed solution was utilized: 2, 6, 10, 14 and 20 mL. The reflux process was carried out at 80 °C for 3 h. The treated samples were then washed with hot water to remove residual acid and base, and the washing process was continued until the pH became close to 7. The neutral rice husk activated carbon (RHAC) samples were then dried at 110 °C for 24 h and cooled in a desiccator.

### 3.3. Characterizations

A Perkin-Elmer infrared spectrometer (FT-IR, Waltham, MA, USA) was used for the investigation of the surface functional groups. The investigated samples were mixed with KBr of spectroscopic grade and made into pellets at a pressure of about 1 MPa. The pellets were about 10 mm in diameter and 1 mm in thickness. The samples were scanned in the spectral range of 4000–400 cm^−1^. X-ray diffraction experiments were performed using a Philips X’pert diffractometer (XRD, JEOL, JSX-60PA, Akishima, Tokyo, Japan) to check the crystalline materials. The analysis was conducted through 2θ values with a range of 10~100° along with low angle, using Cu Kα radiation at a wavelength of λ = 1.54 Å. The surface morphology of the prepared samples was investigated using scanning electron microscopy (SEM, Joel, JSM-IT200, Akishima, Tokyo, Japan) fitted with an energy dispersive X-ray (EDX) for elemental analysis. The topography of the samples was studied using transmission electron microscopy (TEM, Jeol, JEM-100CX II, Akishima, Tokyo, Japan). The surface area and porosity of the prepared samples were assessed using N_2_ adsorption–desorption analysis (Micromeritics ASAP 2020 porosimeter, Norcross, GA, USA). The samples were initially degassed at 110 °C for 10 h under vacuum prior to being analyzed. X-ray photoelectron spectroscopy was utilized to investigate both the bonding states and the chemical compositions of the introduced function materials. XPS data were collected using a K-ALPHA instrument (Themo Fisher Scientific, X-Ray001 400 μm, Waltham, MA, USA) with monochromatic X-ray Al K-alpha radiation −10 to 1350 eV spot size 400 μm at a pressure of 10^−12^ bar with full a spectrum pass energy of 200 eV and at the narrow spectrum of 50 eV.

### 3.4. Ex Situ of the Synthesized Electrodes

Using the E-C lab potentiostat (Bio-Logic Science Instruments SAS, SP-50, Seyssinet-Pariset, France), an electrochemical characterisation has been carried out. A standard three-electrode cell construction was adopted, consisting of a Pt wire counter electrode, an Ag/AgCl reference electrode, and a working electrode made of glassy carbon (D = 3 mm covered with 1 mm Teflon). The electrolyte used for the experiments was a low concentration acid solution (0.1 M H_2_SO_4_), and the sweep potential range used for the tests was 0.5 V to +0.5 V (vs. Ag/AgCl).

## 4. Conclusions

A combined activation agent from phosphoric acid and potassium hydroxide (3.0 M) with a volume ratio of unity shows a distinct performance in activation of a char obtained from the carbonization of rice husk. However, the volume of the activation solution should be optimized, as around 45 mL per gram solid material shows the highest specific capacitance. Compared to the individual activation by the acid and the base, the specific capacitance increases three- and two-fold, respectively, upon utilizing the mixed agent. Although the produced activated carbon contains silicon compounds, the stability in low concentration acid solution (0.1 M H_2_SO_4_) is very distinguishable. Overall, the electrochemical findings imply that the proposed mixed activation agent can be used to make biomass–based hierarchical porous carbon electrode materials with good performance and that have low costs for energy storage.

## Figures and Tables

**Table 1:** Specific capacitance of activated carbons prepared from rice husk using different activation strategies.

Material	Activation	Electrolyte	Sp. Cap. (F/g)	Ref/Year
RH	Thermal 600	3 M KOH	216	[2] 2022
Act-RH–900	NaOH	1 M KOH	150.8	[75] 2021
ACP–1100	KOH	1 M KOH	255	[22] 2020
RS 900 °C was		1 M KCl 1 M KOH	46.79 40.91	[76] 2020
RHPC	NaOH	6 M KOH	263	[38] 2020
RHAC RHAC-T	Thermal/800	1 mol L–1Et4NBF	115 152	[73] 2019
RH	KOH	6 M KOH	315	[31] 2019
Graphene/RH	KOH	1 M Na_2_SO_4_	115	[77] 2017
RH/GR	KOH	1 M (Na_2_SO_4_)	86	[78] 2017
RHPC	NaOH	6 M KOH	97	[9] 2017
RHC	KOH	1 M H_2_SO_4_	225	[79] 2016
RHC 850	KOH	6 M KOH	143	[63] 2016
RH 850	KOH	6 M KOH	147	[63] 2016
RHAC 800	KOH	6 M KOH 1.5 M TEA–BF4	367 147	[80] 2015
RHs	KOH	6 M KOH	250	[81] 2014
RHN–800	NaOH	0.5 M K_2_SO_4_	172.3	[82] 2014
RH	H_3_PO_4_	1 M H_2_SO_4_	112	[83] 2014
Monolayered graphene RH	KOH	1 M H_2_SO_4_	80	[84] 2014
ZnCl_2_/RH	ZnCl_2_	6 M KOH	243	[85] 2013
RH	CO_2_	1 M (Na_2_SO_4_)	112	[86] 2011
RH	H_3_PO_4_/KOH	0.1 M H_2_SO_4_	241.3	This study

## Data Availability

Not applicable.

## Sample Availability

Samples are available from the authors upon request.
